# Functional Asymmetries Routing the Mating Behavior of the Rusty Grain Beetle, *Cryptolestes ferrugineus* (Stephens) (Coleoptera: Laemophloeidae)

**DOI:** 10.3390/insects13080699

**Published:** 2022-08-03

**Authors:** Maria C. Boukouvala, Nickolas G. Kavallieratos, Angelo Canale, Giovanni Benelli

**Affiliations:** 1 Laboratory of Agricultural Zoology and Entomology, Department of Crop Science, Agricultural University of Athens, 75 Iera Odos Str., 11855 Athens, Attica, Greece; 2 Department of Agriculture, Food and Environment, University of Pisa, Via del Borghetto 80, 56124 Pisa, Italy; angelo.canale@unipi.it (A.C.); giovanni.benelli@unipi.it (G.B.)

**Keywords:** copulation, laterality, lateralization, mating success, reproductive behavior, stored-product insect pest

## Abstract

**Simple Summary:**

We evaluated the behavioral asymmetries of *Cryptolestes ferrugineus* (Stephens) (Coleoptera: Laemophloeidae) males during courtship and mating with potential mates. The highest proportion of males showed left-biased approaches towards females, and turned 180° to their left. Right-biased males (i.e., approaching mates from the right and then turning 180°) were fewer than left-biased males. A low percentage of males approaching from the front and back side achieved successful mating. Left-biased-approaching males had a significantly shorter copula duration in comparison with other males. Left-biased males performed shorter copulation attempts and copula in comparison to right-biased males. This research contributes to understand the role of lateralization in the beetle family Laemophloeidae.

**Abstract:**

The rusty grain beetle, *Cryptolestes ferrugineus* (Stephens) (Coleoptera: Laemophloeidae), is a serious secondary pest of stored and processed food commodities. In the present study, we investigated the lateralization of males during courtship and mating, attempting to understand if it can be linked with a high likelihood of successful copulation. Most males exhibited left-biased (41%) approaches towards females, and turned 180° to their left, with 37% mating success. Right-biased males (i.e., approaching from the right and then turning 180°) were fewer than left-biased ones; 26% out of 34% managed to copulate with females. Only 9% out of 13% and 7% out of 11% of the back side- and front side-approaching males succeeded in mating, respectively. Directional asymmetries in approaching a potential mate, as well as the laterality of side-biased turning 180°, significantly affected male copulation success, with left-biased males achieving higher mating success if compared to right-biased males. Copula duration was significantly lower for left-biased-approaching males (1668.0 s) over the others (i.e., 1808.1, 1767.9 and 1746.9 for right-biased, front and back side-males, respectively). Left-biased males performed shorter copulation attempts and copula compared to right-biased males. Overall, our study adds basic knowledge to the lateralized behavioral displays during courtship and copula of *C. ferrugineus*.

## 1. Introduction

Lateralization of the brain (i.e., the different functions and/or structures between the left and right sides of the brain) can increase its ability to perform cognitive tasks involving both hemispheres at the same time [1]. Lateralization has been examined extensively in numerous vertebrate species [2,3,4,5,6,7,8,9,10]. However, invertebrates, especially insects [11,12,13,14], have been relatively little studied. Further research is needed in insects to identify the connection between the mechanisms of neural asymmetries and behavioral traits [15]. Although lateralization has been examined extensively in bees [16,17,18,19,20,21,22], there is a growing tendency to investigate laterality traits in other groups of insects. For instance, concerning Diptera, Benelli et al. [23,24] and Romano et al. [25] examined the lateralized aggressive displays in the Mediterranean fruit fly, *Ceratitis capitata* (Wiedemann) (Diptera: Tephritidae), in the olive fruit fly, *Bactrocera oleae* (Rossi) (Diptera: Tephritidae), and in the blue bottle fly, *Calliphora vomitoria* (L.) (Diptera: Calliphoridae). Benelli and Romano [26] also investigated courtship and mating behavior of the green bottle fly, *Lucilia sericata* (Meigen) (Diptera: Calliphoridae), and the impact of lateralization on these traits during sexual interactions. Behavioral tests on laterality have been carried out on the Asian tiger mosquito, *Aedes albopictus* (Skuse) (Diptera: Culicidae), as well as on the common house mosquito, *Culex pipiens* L. (Diptera: Culicidae) [27,28]. In the case of orthopterans, Romano et al. [29,30] studied the lateralization of the escape and surveillance responses of the migratory locust, *Locusta migratoria* (L.) (Orthoptera: Acrididae), in different instars during biomimetic interactions with a robot predator, while Bell and Niven [31] investigated the forelimb lateralization of the desert locust, *Schistocerca gregaria* (Forsskål) (Orthoptera: Acrididae), during gap crossing. Behavioral asymmetries during courtship and mating have also been examined in the earwigs *Labidura riparia* (Pallas) (Dermaptera: Labiduridae), *Euborellia plebeja* Dohrn, *Nala lividipes* (Dufour) and *Nala nepalensis* (Burr) (Dermaptera: Anisolabididae) [32,33,34,35].

Recent research efforts have focused on the laterality behavior during courtship and mating in species belonging to the order Coleoptera, with a special focus on pests of stored products. For instance, the influence of the geographical origin in relation to rearing media (i.e., a Greek strain reared on wheat, a Greek strain reared on maize and a Peruvian strain reared on maize) on male mating success and lateralization of the rice weevil, *Sitophilus oryzae* (L.) (Coleoptera: Dryophthoridae), has been investigated [36]. Similarly, Boukouvala et al. [37] examined the mating and laterality behavior of strains of the lesser grain borer, *Sitophilus oryzae* (F.) (Coleoptera: Bostrychidae), originating from Greece, Romania, and Turkey. Behavioral observations have been conducted for the tenebrionids, the confused flour beetle, *Tribolium confusum* Jacquelin du Val, the red flour beetle, *Tribolium castaneum* (Herbst) and the yellow mealworm, *Tenebrio molitor* L. (Coleoptera) [38,39,40]. The presence of the lateralized mating traits during courtship and copulation for the khapra beetle, *Trogoderma granarium* Everts (Coleoptera: Dermestidae) [41], and the saw-toothed grain beetle, *Oryzaephilus surinamensis* (L.) (Coleoptera: Silvanidae) [42], has been also studied. In addition, lateralized behavioral traits of both sexes of the larger grain borer, *Prostephanus truncatus* (Horn) (Coleoptera: Bostrychidae), during bio-hybrid intrasexual and intersexual interactions have been investigated [43].

The rusty grain beetle, *Cryptolestes ferrugineus* (Stephens) (Coleoptera: Laemophloeidae), is a serious pest of stored and processed commodities, exhibiting worldwide distribution [44,45,46,47]. Larvae and adults prefer to feed mainly on wheat germ, causing grain damage [48,49,50]. It infests numerous commodities, such as oilseeds, dried tomatoes, black pepper, peanuts, cocoa, coffee bean, dried fruits, chili, hemp, cotton seed, rice, and yams [51,52]. The population growth of this species is affected by the moisture content of the grain, given that it does not develop in very dry grains (i.e., less than 12% moisture content) [50,53]. Females mate repeatedly and their average egg production is about 240 eggs [50]. A thorough analysis of the literature revealed a gap in knowledge related to the lateralization in *C. ferrugineus* males, and more generally in the Laemophloeidae family, during courtship and copulation with potential mates. In a historic study, Rilett [48] described the whole mating sequence of this species. The typical mating of *C. ferrugineus* consists of the male touching the tip of the female’s abdomen while pursuing her. When she stops, the male touches the elytra of the female with his antennae and then he turns and tries to achieve genital contact with the female [48]. In this scenario, the objective of the current study was to quantify the courtship and mating behavior of this beetle pest, and to investigate the presence of population-level behavioral asymmetries characterizing different mating displays of *C. ferrugineus*, shedding light on their potential connection with a high likelihood of mating success.

## 2. Materials and Methods

### 2.1. Insect Rearing and Sex Recognition

Virgin males and females of *C. ferrugineus* were used in our behavioral experiments. Tested individuals were taken from a mass-rearing facility maintained in the Laboratory of Agricultural Zoology and Entomology of the Agricultural University of Athens since 2017. This species was reared in a medium consisting of a mixture of 500 g wheat flour, 20 g yeast and broken wheat kernels on top (thickness of around 1 cm). The preservation of the colonies took place at 30 °C, 65% relative humidity (RH) and in continuous darkness. The initial population of *C. ferrugineus* was obtained from the Institute of Pesticides and Environmental Protection (Belgrade, Serbia). The founding insects were originally collected from Serbian facilities and had been cultured at the same conditions for >18 years.

To obtain virgin male and female adults, pupae were placed separately in 60 mL plastic cups [37]. Sex recognition was conducted at the adult stage, by examining the shape of the mandibles under a stereoscope, according to Rilett [48]. The mandibles of males have a lateral projection near the base, resembling a tooth, while, in females, this projection does not exist. Next, the individuals were kept in the same cups with the addition of 1 g of the rearing medium until the beginning of the tests.

### 2.2. Behavioral Observations

Adults of *C. ferrugineus* mate within one or two days after their emergence [48]. Thus, all tested individuals were <1 day old. According to earlier behavioral studies on various stored-product insect species [37,38,39,40,41,42], before the beginning of the experiments, we exposed separately males and females for 3 h to natural conditions of light, as described by Romano et al. [36], Boukouvala et al. [37,39,40,41] and Benelli et al. [38,41]. The observations were carried out under natural photoperiod conditions. The type of arena that was used in the trials followed Boukouvala et al. [42]. Mating trials were realized between 11 am and 7 pm, at 30 °C and 60% RH [36,37,38,39,40,41,42].

A virgin male and a virgin female of *C. ferrugineus* were transferred into the testing arena, to investigate the presence of lateralized population-level behavioral asymmetries during courtship [37]. We also described and quantified the courtship and mating behavior sequence of this species. The evaluation was carried out visually for a period of 60 min by an individual, or till the termination of the sexual activity, if there was any [38]. We recorded the duration of the following phases: (i) mate recognition (i.e., time spent by the male to detect the female), (ii) precopula (i.e., time spent by the male chasing the female, until she stopped, and time spent by the male to achieve genital contact with the female), (iii) copula (i.e., from the male insertion of the aedeagus into the female genital chamber until genital disengagement) and (iv) the duration of the whole courtship and mating sequence [36,37,38,39,40,42].

We observed a total of 262 mating pairs of *C. ferrugineus*, of which 123 were not taken into account in statistical analysis because (i) the pair remained immobile or did not have any sexual interaction during the 60-min observation [38]; (ii) the females were close to the walls of the arena, influencing the laterality observations and obstructing the directional approaches of the males [36], and (iii) females did not stop during chasing and males gave up the attempt. Thus, 139 mating pairs were considered for behavioral analysis. Furthermore, 111 (out of 139) mating pairs exhibited successful copulations; these data were analyzed for assessing the possible impact of the lateralized approach of females or lateralized male turning 180° to achieve genital contact on the duration of the *C. ferrugineus* main mating traits. As has been reported in previous studies on other stored-product beetle pests [37,40,42], and according to preliminary observations, it was not necessary to add any food source in the arena for the mating of *C. ferrugineus*, thus avoiding the possible bias of the orientation of the tested males.

### 2.3. Statistical Analysis

Data concerning the mating success of *C. ferrugineus* performing or not a lateralized approach towards potential mates, or exhibiting lateralized turning 180° before genital contact with a female, were analyzed with the JMP 16.2 software [54], using a weighted generalized linear model with binomial distribution: *y* = *Xβ* + *ε*, where *y* is the vector of the observations (i.e., successful or unsuccessful copulation), *X* is the incidence matrix, *β* is the vector of fixed effect (i.e., the approached side of the female’s body, or the left or right turning 180° of males) and *ε* is the vector of the random residual effect. A probability level of 0.05 was used for the significance of differences between values.

The effect of orientation in approaching the female and the lateralized turning of 180° of males on the duration of the main behavioral mating traits, i.e., mate recognition, precopula (chasing and copulation attempt) and copula, were not normally distributed; they were analyzed by the Steel–Dwass test, with α = 0.05 [37,42].

## 3. Results

**Figure 1:**
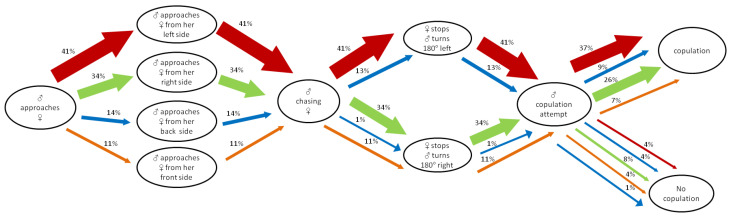
Flow chart showing population-level behavioral asymmetries during courtship and mating of *Cryptolestes ferrugineus*. Red, green, blue and orange arrows indicate the body side of females (left, right, back and front, respectively) approached by males. Red and green arrows also indicate the lateral turn of males before the genital contact. The thickness of each arrow indicates the proportion of beetles displaying each behavior (*n* = 139 mating pairs).

The main lateralized traits of *C. ferrugineus* males during the mating sequence are presented in Figure 1. Most males exhibited left-biased approaches towards females. In our tests, 41% of males approached females from their left side and turned 180° to their left, where 37% of males copulated successfully and only 4% of them failed to mate. Right-biased males (i.e., approaching and turning 180°) followed, where 26% out of 34% managed to copulate with females and 8% did not mate. Fewer *C. ferrugineus* (14%) approached the females from their back side, where 13% of males turned 180° to the left side, and 9% (out of 13%) of them succeeded in mating. However, only 1% of the back side-males chose to turn 180° from the right side, leading to successful copulation. A rate 11% of males approached the females from the front side and performed a 180° turn from their right, among which 7% (out of 11%) of them managed to copulate.

**Figure 2:**
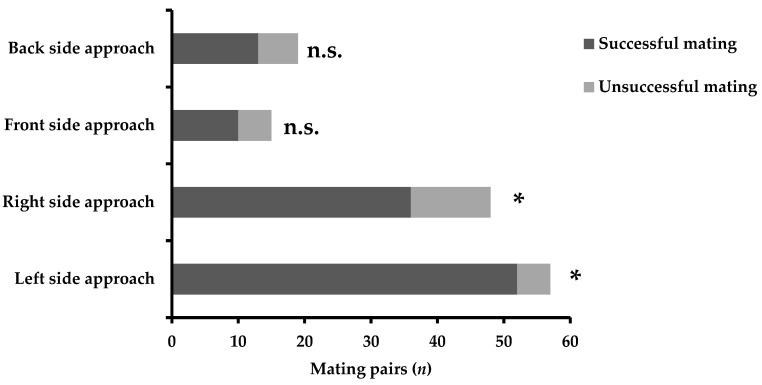
Male mating success of *Cryptolestes ferrugineus* exhibiting or not exhibiting lateralized approaches towards females. The asterisk indicates significant differences; n.s. = not significant (generalized linear model, binomial distribution, *p* < 0.01).

**Figure 3:**
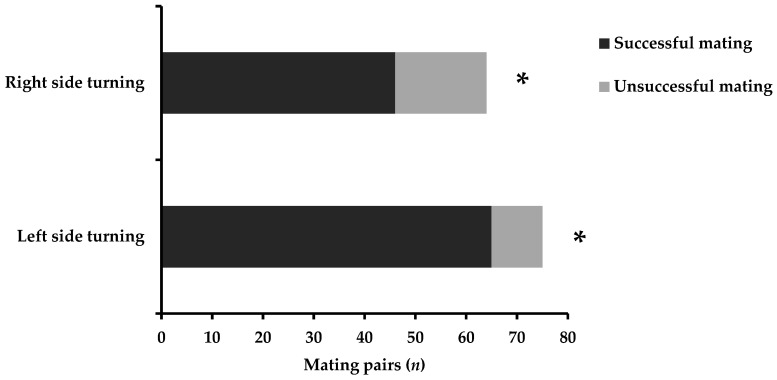
Male mating success of *Cryptolestes ferrugineus* performing lateralized turning before genital contact with female. The asterisk indicates significant differences; (generalized linear model, binomial distribution, *p* < 0.01).

The mating success of *C. ferrugineus* males exhibiting or not exhibiting asymmetric approaches is presented in Figure 2. Side biases when approaching females significantly affected the copulation success of males (*χ*^2^ = 9.002, DF = 3, *p* < 0.001). Left-biased approaches of males led to higher mating success (*χ*^2^ = 38.772, DF = 1, *p* < 0.001), followed by the right-biased males (*χ*^2^ = 12.021, DF = 1, *p* < 0.001). The successful copulation was lower for back side- or front side-approaching males (*χ*^2^ = 2.632, DF = 1, *p* > 0.05 and *χ*^2^ = 1.733, DF = 1, *p* > 0.05 for back side and front side, respectively). Concerning the successful copulation of males performing lateralized turning 180° before the genital contact, results are shown in Figure 3. The laterality of side-biased turning 180° influenced the mating success of males (*χ*^2^ = 4.778, DF = 1, *p* < 0.001). Males that preferred to turn 180° from their left side displayed higher copulation success, i.e., 65 pairs mated successfully (*χ*^2^ = 40.347, DF = 1, *p* < 0.001), compared to males that turned 180° from their right side, i.e., 46 pairs mated successfully (*χ*^2^ = 12.266, DF = 1, *p* < 0.001).

Mate recognition duration in left-biased males (322.3 s) was lower than the duration characterizing right-biased males (410.7 s). However, the duration of approaches of males that approached females from their front side (166.1 s) was significantly lower than approaches from the left and right sides (Table 1). Regarding back side-approaching males (189.7 s), the duration did not differ significantly from the durations of left-biased and front side-approaching males. During precopula, the front side- and back side-approaching males (152.9 and 116.3 s for front side- and back side-approaching males, respectively) spent significantly more time chasing the females if compared to the left- and right-biased males (86.5 and 81.0 s for left- and right-biased males, respectively). During copulation attempts, no significant differences were noted among the males that performed or not lateralized approaches. However, the duration of copula was significantly lower for the left-biased males (1668.0 s) if compared to other males (i.e., 1808.1, 1767.9 and 1746.9 for left-biased, front side and back side-males, respectively). Duration of mate recognition and chasing did not differ significantly between males that turned 180° from their right or left side (Table 2). Significant differences were noted in the duration of the copulation attempt and copula among left- and right-biased males (i.e., 33.7 and 1683.8 s for copulation attempt and copula of left-biased males vs. 40.1 and 1799.3 s for copulation attempt and copula of right-biased males). Left-biased males performed shorter copulation attempts and copula than the right-biased males (Table 2).

## 4. Discussion

Our findings add knowledge on the role of behavioral asymmetries in the courtship and mating of *C. ferrugineus*, an important pest of stored and processed food commodities worldwide. The results of the present study indicate that males of this species performed left-biased mate approaching and turned 180° from their left side, achieving higher mating success over right-biased males. The success of left-biased males could be attributed to differences between males and females in morphology, i.e., males have longer antenna, the heads of males are larger, the thorax is wider in males, the tarsi of males are 5-5-4 vs. 5-5-5 in females [48]. For instance, Eberhard [55] reported that the morphology of the abdominal sternites affected the courtship of *Phyllophaga* spp. (Coleoptera: Melolonthidae). Further research is needed to shed light on this issue. Of note, earlier behavioral studies on other stored-product pests belonging to the order Coleoptera revealed that left-biased males achieved higher mating rates over right-biased males. For instance, males of *T. confusum* and *T. castaneum* that performed a left-biased approach during courtship were more successful than right-biased males [38,39]. Moreover, males of *T. molitor* achieved higher proportions of successful copulations when they moved on the apex of the female abdomen and attempted to mount from the left side in comparison to the right side. Benelli et al. [38] reported that male *S. oryzae* performed left-biased copulation attempts towards potential mates, exhibiting a periodical right-biased head wagging behavior, regardless of the fact that most of the males had previously attempted left-biased copulations. The same trend has been reported for *T. granarium* males. They exhibited left-biased asymmetries during the recognition approach of females, resulting in more successful mating if compared to the right-biased males [41]. Taking into account the above-mentioned studies, it seems that there is an abundance of left-biased courtship and mating traits in stored-product beetle species. However, there are also two studies with contrasting results. Strains of *R. dominica* originating from three distinct geographical sites (i.e., Greece, Romania, and Turkey) have been found to be right-biased when attempting to mate, resulting in a higher percentage of successful copulations over left-biased and back side-approaching males [37]. Similarly, males of *O. surinamensis* performed mounting attempts from the right side of females, achieved higher mating success over left-biased males [42].

One of the most important findings of the present study is the impact of male laterality behavior (e.g., left or right turning 180°) on the duration of each phase during courtship and copulation. Concretely, the duration of mate recognition and chasing was lower in left-biased males in comparison to the right-biased ones, while the duration of copulation attempts and copula was significantly shorter for the left-biased males than the right-biased males. The high proportion of successful matings by *C. ferrugineus* left-biased males, combined with the shorter duration of each phase, may positively affect the development of large insect populations. Male laterality could be partially explained by the higher abundance of sensilla on a certain side of the female head that create olfactory and tactile side biases, as has been reported for Hymenoptera [19,56]. Whether this is a case for stored-product Coleoptera remains to be confirmed. This knowledge can be a useful tool to optimize mass-rearing techniques by choosing behavioral traits as indicators of male quality over time [37]. Therefore, considering the knowledge obtained so far for the mating and laterality behavior of key stored-product insect pests, the selection of the left-biased males of *S. oryzae*, *T. granarium*, *T. confusum*, *T. castaneum* and *T. molitor*, as well as the right-biased males of *R. dominica* and *O. surinamensis*, may be useful to initiate laboratory mass rearing that can develop quickly. However, further experimentation is needed to investigate this issue, especially the behavioral analysis of populations from different geographical sites, given that origin affects lateralization [36,37]. On the basis of previous research and the current study, we are able to propose the following order of species presenting left-biased asymmetries during courtship and mating: *C. ferrugineus* (1683.8 s) > *T. confusum* (~420 s) > *S. oryzae* (~140 s) > *T. castaneum* (~130 s) > *T. molitor* (84.5 s) > *T. granarium* (65.83 s) [38,39,40,41]. Therefore, when multiple options are available for conducting experiments (e.g., insecticide development and evaluation, bioecology) with stored-product insect species, *C. ferrugineus* would be a less favorable selection, because it exhibits much longer copulation in relation to other stored-product insects. However, whether *C. ferrugineus* can be used as a model species in science, such as *Tribolium* spp. [38,39] and *T. molitor* [40], merits further investigation.

The behavioral trait of males turning 180° before genital contact with the female is also evident in a closely related species, the flat grain beetle, *Cryptolestes pusillus* (Schönherr) (Coleoptera: Laemophloeidae) [48]. Based on our previous work [36,37,38,39,40,42], this characteristic is not common among other stored-product coleopteran species, since malemounts the female to execute genital contact. For instance, *T. castaneum*, *T. confusum*, *T. molitor*, *O. surinamensis*, *R. dominica* and *S. oryzae* conduct mounting attempts and remain upon females during copulation. An exception is represented by *T. granarium*, which shows 45°, 90° and, rarely, 180° turns [41]. Even in this case, the 180° turning is performed by 3%, while 45° and 90° correspond to 46 and 27% of the tested males [41]. The different ways in which males mount females might be due to anatomical or morphological differences among the different species. Whether the aforementioned behavior affects the life history of *C. ferrugineus* needs additional research.

## 5. Conclusions

Our findings indicate significant differences in the success of copulation among the left- and right-biased approaching males, and among males with left- or right-biased turning 180°. More experiments should be carried out to shed light on the complex phenomenon of lateralization to clarify the mechanisms that affect the mating behavior of beetle pests of stored products. Although our research has included some noxious stored-product coleopterans, it has been conducted per species [38,39,40,41,42] or strain [36,37]. However, in the storage environment, numerous species co-exist [47], an issue that alters the damaging potential of the co-existing species [57]. Whether laterality is also affected when different species grow on the same substratum is a challenging new area for behavioral investigations.

## Figures and Tables

**Table 1:** Effect of the recognition side bias on the duration of *Cryptolestes ferrugineus* main mating traits. Values are means followed by standard errors (SE). Within each column, means followed by different letters are significantly different (Steel–Dwass test, *p* < 0.05). Where no letters exist, differences are not significant among side-biased parameters.

		Precopula		
Laterality	Mate Recognition (s)	Chasing (s)	Copulation Attempt (s)	Copula (s)
Left-biased	322.3 ± 21.4 ab	86.5 ± 2.9 c	32.4 ± 11.4	1668.0 ± 16.8 b
Right-biased	410.7 ± 40.4 a	81.0 ± 3.2 c	39.1 ± 7.4	1808.1 ± 13.3 a
Front side	166.1 ± 7.4 c	152.9 ± 4.8 a	38.0 ± 13.9	1767.9 ± 12.4 a
Back side	189.7 ± 22.9 bc	116.3 ± 4.7 b	41.8 ± 12.1	1746.9 ± 2.6 a
*χ* ^2^	30.12	54.33	10.14	53.02
DF	3	3	3	3
*p*-value	<0.0001	<0.0001	0.3194	<0.0001
Tested beetles (*n* = left + right + front + back-biased)	57 + 48 + 15 + 19 = 139	57 + 48 + 15 + 19 = 139	57 + 48 + 15 + 19 = 139	52 + 36 + 10 + 13 = 111

**Table 2:** Duration of *Cryptolestes ferrugineus* main mating behavior displays during sexual interactions characterized by a lateral turn of 180°. Values are means followed by standard errors (SE). Within each column, asterisks indicate significant differences (Steel–Dwass test, *p* < 0.05). Where no asterisks exist, no significant differences were noted.

		Precopula		
Laterality	Mate Recognition (s)	Chasing (s)	Copulation Attempt (s)	Copula (s)
Left-biased	285.7 ± 18.1	94.2 ± 2.9	33.7 ± 9.1	1683.8 ± 14.0
Right-biased	355.5 ± 33.7	97.8 ± 4.7	40.1 ± 6.5	1799.3 ± 11.0
*χ* ^2^	2.14	0.01	29.7	46.93
DF	1	1	1	1
*p*-value	0.1431	0.9865	<0.0001 *	<0.0001 *
Tested beetles (*n* = left + right-biased)	75 + 64 = 139	75 + 64 = 139	75 + 64 = 139	65 + 46 = 111

## Data Availability

Data are contained within the article.
